# The association between acetylsalicylic acid and subarachnoid haemorrhage: the Framingham Heart Study

**DOI:** 10.1038/s41598-023-33570-9

**Published:** 2023-04-21

**Authors:** Frederick Ewbank, Jacqueline Birks, Diederik Bulters

**Affiliations:** 1 Department of Neurosurgery, University Hospital Southampton, Southampton, UK; 2 Centre for Statistics in Medicine, University of Oxford, Oxford, UK; 3 Department of Neurosurgery, University Hospital Southampton, Southampton, UK

**Keywords:** Neurology, Neurological disorders

## Abstract

Studies investigating the association between acetylsalicylic acid (ASA) use and spontaneous subarachnoid haemorrhage (SAH) in the general population have produced conflicting results. The aim of this study is to clarify the relationship between SAH and ASA. We included all participants who reported on ASA use during interim examinations of the Framingham Heart Study Cohorts. Using Cox proportional-hazards regression modelling, we estimated the hazard ratio (HR) associated with ASA use. 7692 participants were included in this study. There were 30 cases of SAH during follow up, with an estimated incidence of 10.0 per 100,000 person- years (CI 6.90–14.15). Univariate analysis showed no association between regular ASA use and SAH (HR, 0.33 [0.08–1.41]; *p* = 0.14). This was similar when accounting for smoking (HR, 0.35 [0.08–1.51]; *p* = 0.16). Using a large longitudinal dataset from the Framingham Heart Study, we observed some evidence suggesting fewer SAH in those participants taking regular ASA. However, multivariate statistical analysis showed no significant association between ASA use and SAH. Due to the low incidence of SAH in the general population, the absolute number of SAH events was low and it remains uncertain if a significant effect would become apparent with more follow up.

## Introduction

Subarachnoid haemorrhage (SAH) is a rare form of stroke with an incidence between 6 and 10 per 100,000-person years^1,2^. Despite only accounting for 5% of all strokes, SAH is associated with substantial morbidity and mortality^3–6^. Rupture of intracranial aneurysms accounts for 85% of non-traumatic SAH cases. The prevalence of unruptured aneurysms is comparatively high at 3.2%, and consistent with the observation that rupture rates for unruptured aneurysms are relatively low^7^. Currently, neurosurgical or endovascular approaches are the only prophylactic treatment of unruptured intracranial aneurysms. Given these treatments carry their own risks, many cases are managed conservatively. Despite advances in our understanding of the pathophysiology of intracranial aneurysms, there are no other therapeutic options.

Acetylsalicylic acid (ASA) is a well-established antiplatelet agent and known to increase the risk of bleeding, including predisposing to intracerebral haemorrhage^8–11^. However, the pathophysiology of aneurysmal SAH is different. Inflammation is believed to play an important role in the formation and rupture of intracranial aneurysms. It has been shown in animal models that the COX-2/ mPGES/ PGE-2E pathway is central to this and can be attenuated by ASA. Therefore, it has been proposed that ASA may provide protection from SAH^12–14^.

Previous research in this area has produced conflicting results^15–17^. It is difficult to interpret and generalise studies due to marked differences in study populations, study designs and definitions of SAH. Specifically, studies including cohort and case- control studies, patients with and without known aneurysms, and SAH of all causes and aneurysmal SAH. Consistent with this, our meta-analysis showed a high degree of heterogeneity between studies especially between studies of patients with aneurysms versus those in the general population^18^. This heterogeneity was reduced when considering studies of patients with unruptured aneurysms where an inverse relationship between ASA use and SAH was observed, although a preponderance of case- control studies with higher risk of bias was noted^19–24^. Studies conducted in a general population without prior diagnosis of aneurysm produced conflicting results, with some suggesting benefit, some no relationship and others suggesting a negative association^18,25–30^.

In view of this conflicting data, we set out to further study the relationship between ASA and SAH in a large prospectively studied cohort of participants without a prior diagnosis of an intracranial aneurysm with longitudinal follow up from the Framingham Heart Study.

## Methods

The Framingham Heart Study was established in 1948 as a longitudinal, community- based, population study aimed at investigating all forms of cardiovascular disease including stroke. Between 1948 and 1950, participants aged between 28 and 62 were enrolled in this study and subsequently underwent biennial examinations. Between 1971 and 1974, children of the Original Cohort were enrolled in a separate Offspring Cohort. Details of the study design, implementation and criteria for diagnosis have been previously published^31,32^. This study is approved by the Institutional Review Board (Ethics and Research Governance Online (ERGO), University of Southampton: 54,198) and the National Heart, Lung, and Blood Institute, National Institutes of Health, Department of Health and Human Services (ID 8194). Written informed consent was obtained from participants, including guardians or parents where appropriate. Research was performed in accordance with STROBE guidelines and the Declaration of Helsinki.

We included all participants who reported on ASA use during interim examinations. ASA use was reported at examinations 13 and examinations 17- 32 in the Original Cohort. ASA use was reported at examinations 2–8 in the Offspring Cohort. Due to variations in the reporting of dosing and frequency at different examinations, we defined ASA use as regular ASA use (at least one tablet of ASA per day). We also performed a sensitivity analysis defining ASA use as any ASA use (where all participants taking ASA were included irrespective of frequency). In instances when censoring or a SAH event occurred prior to the first report of ASA use it was assumed that the ASA use in the preceding time period was the same as the subsequent report.

Stroke events were detected by review of interim examinations, surveillance of all admissions to the local hospital, and scrutiny of outside hospital records, including autopsy reports. Suspected strokes were referred to the Framingham Heart Study for detailed examination. The diagnosis of cerebrovascular disease was based on the occurrence of a clinically evident stroke documented by clinical records reviewed by two neurologists. Stroke was defined as the sudden or rapid onset of a focal neurologic deficit persisting for greater than 24 h. The diagnosis of SAH was based on a history suggestive of this process such as abrupt onset headache, with or without change in the level of consciousness, and signs of meningeal irritation with or without other localizing neurological deficits. Traumatic SAH was excluded based on the clinical history. Older SAH cases in the Original Cohort were diagnosed based on the combination of clinical syndrome and cerebrospinal fluid analysis. More recent cases in the Original Cohort and those cases in the Offspring Cohort also used imaging to confirm SAH. The proportion of aneurysmal SAH cases is unknown and other types of nontraumatic SAH, such as perimesencephalic SAH, cannot be excluded. This study followed the STROBE guidelines for reporting observational studies.

### Statistical analysis

Data from the Original Cohort and Offspring Cohorts were combined. A Cox proportional-hazards regression modelling stratified by cohort was used to estimate the hazard ratio (HR) associated with ASA use. Proportional hazards assumption was checked using graphical diagnostics based on the scaled Schoenfeld residuals. ASA use, age, smoking status and blood pressure were included as time varying covariates. Univariate and multivariate analyses were conducted. Multiple imputation was used to account for missing variables in the multivariate analysis. A stepwise variable selection procedure including forward and backward iterations was used to obtain the best candidate final regression model for the multivariate analysis. The significance levels for entry (SLE) and for stay (SLS) equalled 0.15. Statistical analysis was conducted in R (version 4.0.5) and Stata (version 15.1). 

## Results

A total of 7692 participants out of the total population of 10,082 participants in the Framingham Original and Offspring Cohorts reported sufficient information on ASA usage for inclusion in this study. 3140 participants originated from the Original Cohort, while 4552 participants originated from the Offspring Cohort. Overall, there were a total of 47,321 observations, over a total 298,790 years of follow up. Information on ASA use was available for 47,278 observations. Median follow up for each participant was 41 years (range 0.2–68.1). Participants reported taking any form of ASA for a total of 123,488 years of the total 298,790 years of follow up, and of those taking ASA, participants reported taking regular ASA for 51,938 years. There were 30 SAH cases reported over this time period. Baseline characteristics are shown in Table 1.

**Table 1:** Descriptive statistics of baseline variables included in this study. *SD* Standard deviation.

Variables		Original cohort (n = 3140)		Offspring cohort (n = 4552)	
		Mean (± SD)/ %	No. of participants (% missing)	Mean (± SD)/ %	No. of participants (% missing)
Age (years)		66.4 ± 8.2	3140 (0%)	45.1 ± 10.4	4552 (0%)
Males		41.8%	3140 (0%)	48.2%	4552 (0%)
Smoking	Current smoker	26.5%	3140 (0%)	36%	4452 (0%)
	History of smoking	40.0%		48.2%	
	No. cigarettes per day if current smoker	18.6 ± 11.8		23.1 ± 13.4	
Blood pressure	Hypertension	26.4%	3128 (0.4%)	11.7%	4548 (< 0.1%)
	Diastolic blood pressure (mmHg)	78.9 ± 10.5	3138 (< 0.1%)	78.3 ± 9.8	4551 (< 0.1%)
	Systolic blood pressure (mmHg)	139.5 ± 21.2	3138 (< 0.1%)	123 ± 17.2	4552 (0%)
Diabetes mellitus		6.4%	3137 (0.1%)	1.4%	4548 (< 0.1%)
Hyperlipidaemia		2.1%	3137 (0.1%)	1.2%	4550 (< 0.1%)
Aspirin use	Any aspirin use	62.9%	3139 (< 0.1%)	25.7%	4552 (0%)
	Regular aspirin use	9.0%		3.7%	
Mean follow up (years)		40.8 ± 11.0		37.5 ± 8.8	
Total follow up (years)		128,175		170,609	

**Figure 1:**
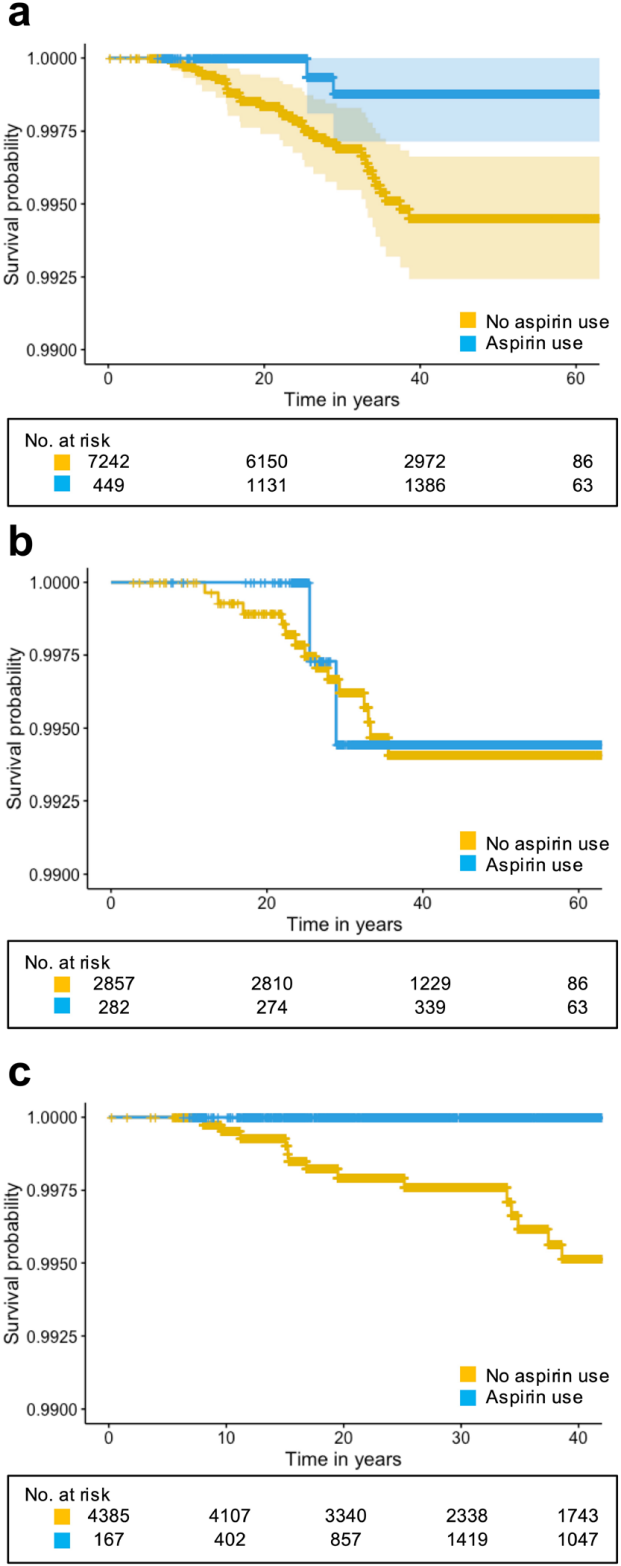
Kaplan–Meier plot comparing the rates of SAH in participants reporting regular aspirin use and those not. (**a**) All participants included in this study. (**b**) All participants included in the original cohort. (**c**) All participants included in the offspring cohort.

The incidence of SAH in this study was 10.0 per 100,000 person- years (CI 6.90–14.15). The estimated incidence of SAH in those participants not currently taking regular ASA was 11.3 per 100,000 (CI 7.70–16.20) (Fig. 1). The estimated incidence of SAH in those participants currently taking regular ASA was 3.9 per 100,000 person years (CI 0.65–12.72) (Fig. 1). For those participants suffering from SAH, regular ASA accounted for 16% of the follow up period. For those participants not suffering from SAH, regular ASA use accounted for 21% of the follow up period.

The results of univariate analysis are shown in Table 2. There was no significant relationship between regular ASA use and SAH (HR, 0.33 [0.08–1.41]; *p* = 0.14). Considering the individual cohorts, there was no significant effect of regular ASA in the Original Cohort (HR, 1.09 [0.24–4.90]; *p* = 0.91). No participants suffered SAH when taking ASA regularly in the Offspring Cohort, precluding formal estimates of a hazard ratio. Smoking was significantly associated with increased risk of SAH (HR, 2.76 [1.30–5.86]; p < 0.01). Univariate analysis showed that all forms of ASA use was not associated with SAH (HR, 0.74 [0.38–1.63]; *p* = 0.46).

**Table 2:** Univariate and multivariate regression analysis stratified by cohort. *HR* Hazard ratio; *CI* confidence interval.

Variables		Univariate		Multivariate	
		HR (CI)	*P* value	HR (CI)	*P* value
Aspirin use	All forms of aspirin use	0.74 (0.38–1.63)	0.46	–	–
	Regular aspirin use	0.33 (0.08–1.41)	0.14	0.35 (0.08–1.51)	0.16
Age		0.98 (0.94–1.02)	0.34	–	–
Sex (male)		0.66 (0.31–1.42)	0.29	–	–
Current smoker		2.76 (1.30–5.86)	< 0.01	2.70 (1.27–5.73)	0.01
Blood pressure	Hypertension	1.20 (0.56–2.58)	0.64	–	–
	Mean arterial blood pressure (/mmHg)	1.01 (0.98–1.04)	0.36	–	–
	Systolic blood pressure (/mmHg)	1.01 (0.99–1.03)	0.34	–	–
	Diastolic blood pressure (/mmHg)	1.01 (0.98–1.05)	0.49	–	–
Diabetes mellitus		0.54 (0.07–3.98)	0.55	–	–
Hyperlipidaemia		0.64 (0.13–3.09)	0.58	–	–

ASA use and smoking were included in the multivariate analysis. The results of multivariate analysis are shown in Table 2. Multivariate analysis showed similar results for ASA, when accounting for smoking status (HR, 0.35 [0.08–1.51]; *p* = 0.16). Smoking remained the only variable significantly associated with SAH (2.70 [1.27–5.73]; *p* = 0.01). A sensitivity analysis performed accounting for other known risk factors, including sex, age, and hypertension, reaffirmed these findings (0.34 [0.07–1.48]; *p* = 0.15).

## Discussion

The relationship between ASA use and SAH remains controversial, and we set out to investigate this further. Using a large longitudinal dataset from the Framingham Heart Study, we observed some evidence suggesting fewer SAH in those participants taking regular ASA. However, while you would expect the anti-inflammatory effects of ASA to counteract the effects of smoking, multivariate statistical analysis showed no significant association between ASA use and SAH.

Only two studies have conducted survival analysis investigating the association between ASA use and SAH^19,24^. Both studies were conducted in a population of patients with known aneurysms and reported a protective association between ASA and SAH. This study is the first to conduct survival analysis in a population not previously known to have an aneurysm. Despite not reaching significance these results could still be consistent with the effects of ASA being diluted, as only 3% of the general population might be expected to have unruptured aneurysms^33^. However, the number of SAH events in this study were similar or larger making this less likely.

There were also some differences between the Original and Offspring Cohorts. While it was not possible to generate hazard ratios for the Offspring Cohort, it was notable that there were no events in those participants taking regular ASA at all. This could be a chance observation related to the lower use of ASA in the Offspring Cohort. However, there are other differences in the characteristics of each of the cohorts and importantly as the indications for ASA use have changed over decades it is unlikely many patients in the Original Cohort took regular ASA for the prevention of cardiovascular disease.

### Strengths

Many features of the Framingham Heart Study make it ideally suited for studying the association between SAH and ASA. Crucially the Framingham Heart Study conducts regular serial follow up of participants. Unlike a number of previous studies, accurate follow up of key variables, such as smoking, ensures appropriate control of confounding factors. This allowed us to conduct time varying analyses to more accurately clarify the role of ASA. It is likely that insufficient follow up of ASA use in previous studies has led to confounding bias. Further to this, thorough assessment and validation of SAH cases using hospital and clinic records by two neurologist ensures the validity of SAH cases. Other studies have often provided little or no detail of how cases were defined or used surrogate criteria, such as admission to neurological centre, which would underestimate the total number of cases. The incidence of SAH in this study is on the higher end of the expected range^1,2^. However, this is consistent with the dates of enrolment (1948–1974), given the observation that SAH rates in 1980 are reported to be 10.2 (8.4–12.5) per 100,000 person years^1^. This is presumed to be due to a higher prevalence of smoking and lower rates of preventative repair of unruptured intracranial aneurysms.

### Limitations

Despite the size of the Framingham Heart Study and length of follow up, SAH is a rare event and as discussed, the number of cases in this study remains low. It is estimated that approximately 50 events would be required to achieve significance in the cohort. Furthermore, despite the strength of this study being that all SAH cases were validated by two neurologists, cases in this cohort are not defined as aneurysmal or non-aneurysmal. While the majority are likely to be aneurysmal, it is also likely that a number of SAH cases in this study are non-aneurysmal as in many previous studies. There is no hypothetical or mechanistic argument for non-aneurysmal SAH being related to ASA. Given the antiplatelet effects of ASA and its association with intracranial haemorrhage, it may be expected to increase the risk of non-aneurysmal SAH^8–10^. In a study not distinguishing between SAH types, ASA could reduce the incidence of aneurysmal SAH in some patients but increase the incidence of non-aneurysmal SAH in other patients masking an effect in patients with aneurysms. This would be in keeping with the findings of our meta-analysis where we observed a relationship between ASA and SAH in patients with known aneurysms but not in the general population^18^.

Another limitation is the inability to comprehensively account for SAH resulting in sudden deaths occurring outside hospital, subsequently underestimating the incidence of SAH . This is inherent in any study of SAH. However, the longitudinal design of the Framingham Heart Study and the review of the records for all deaths and autopsy reports, should have minimised this.

The assumption that ASA use in the preceding time period was the same as the subsequent report when censoring or a SAH event occurred prior to that report may not have held in all cases. Based on the dates of enrolment, it is unlikely that any participants underwent endovascular treatment of the aneurysm and hence it is unlikely that any participants would be started on ASA for this reason. It is possible that participants were more likely to be taking ASA as analgesia following a SAH or less likely to take ASA after SAH due to a perceived concern taking an antiplatelet agent after a bleed. It is therefore possible this diluted a relevant difference which consequently didn’t reach significance.

Finally, the inability to differentiate between different doses of ASA and their frequency could have influenced the results. The rates of ASA use, and especially any ASA use, were greater in the Original Cohort. This can be expected given the dates these participants were enrolled and their age at inclusion. These participants are more likely to have taken ASA for pain relief or inflammation and more likely to have taken greater doses (300/325 mg), whereas the Offspring Cohort are more likely to be taking prophylactic low dose ASA (75/81 mg) for primary or secondary prevention against cardiovascular or cerebrovascular disease. Therefore, this pattern makes it unlikely that any relationship was missed due to a dose relationship given it was the Offspring Cohort in which no SAH events occurred in the ASA group.

## Conclusion

Data from the Framingham Heart Study does not clearly show a relationship between regular ASA use and SAH in the general population. Due to the low incidence of SAH in the general population, the absolute number of SAH events was low and it remains uncertain if a significant effect would become apparent with more follow up. However, these results suggest a negative relationship, as is seen with other types of cerebral haemorrhage, is unlikely.

## Supplementary Information

Supplementary Information.

## Data Availability

The datasets generated and/or analysed during the current study are available in the Biologic Specimen and Data Repository Information Coordinating Centre, National Heart, Lung, and Blood Institute, National Institutes of Health, Department of Health and Human Services, US. https://biolincc.nhlbi.nih.gov/studies/framcohort/. https://biolincc.nhlbi.nih.gov/studies/framoffspring/.

## References

[1] de Rooij NK, Linn FH, van der Plas JA, Algra A, Rinkel GJ (2007). Incidence of subarachnoid haemorrhage: A systematic review with emphasis on region, age, gender and time trends. J. Neurol. Neurosurg. Psychiatry.

[2] Etminan N (2019). Worldwide incidence of aneurysmal subarachnoid hemorrhage according to region, time period, blood pressure, and smoking prevalence in the population: A systematic review and meta-analysis. JAMA Neurol..

[3] Gonzalez-Perez A, Gaist D, Wallander MA, McFeat G, Garcia-Rodriguez LA (2013). Mortality after hemorrhagic stroke: Data from general practice (the health improvement network). Neurology.

[4] Longstreth WT, Nelson LM, Koepsell TD, van Belle G (1993). Clinical course of spontaneous subarachnoid hemorrhage: A population-based study in King County Washington. Neurology.

[5] Fogelholm R, Hernesniemi J, Vapalahti M (1993). Impact of early surgery on outcome after aneurysmal subarachnoid haemorrhage. A population-based study. Stroke.

[6] Stegmayr B, Eriksson M, Asplund K (2004). Declining mortality from subarachnoid hemorrhage: changes in incidence and case fatality from 1985 through 2000. Stroke.

[7] Thompson BG (2015). Guidelines for the management of patients with unruptured intracranial aneurysms: A guideline for healthcare professionals from the American heart association/American stroke association. Stroke.

[8] McQuaid KR, Laine L (2006). Systematic review and meta-analysis of adverse events of low-dose aspirin and clopidogrel in randomized controlled trials. Am. J. Med..

[9] Whitlock EP, Burda BU, Williams SB, Guirguis-Blake JM, Evans CV (2016). Bleeding risks with aspirin use for primary prevention in adults: A systematic review for the US preventive services task force. Ann. Intern. Med..

[10] Heavey DJ, Barrow SE, Hickling NE, Ritter JM (1985). Aspirin causes short-lived inhibition of bradykinin-stimulated prostacyclin production in man. Nature.

[11] Boullin DJ, Bunting S, Blaso WP, Hunt TM, Moncada S (1979). Responses of human and baboon arteries to prostaglandin endoperoxides and biologically generated and synthetic prostacyclin: Their relevance to cerebral arterial spasm in man. Br. J. Clin. Pharmacol..

[12] Caird J, Napoli C, Taggart C, Farrell M, Bouchier-Hayes D (2006). Matrix metalloproteinases 2 and 9 in human atherosclerotic and non-atherosclerotic cerebral aneurysms. Eur. J. Neurol..

[13] Tulamo R (2010). Lack of complement inhibitors in the outer intracranial artery aneurysm wall associates with complement terminal pathway activation. Am. J. Pathol..

[14] Chalouhi N (2012). Biology of intracranial aneurysms: Role of inflammation. J Cereb Blood Flow Metab..

[15] Phan K, Moore JM, Griessenauer CJ, Ogilvy CS, Thomas AJ (2017). Aspirin and risk of subarachnoid hemorrhage: Systematic review and meta-analysis. Stroke.

[16] Qian C, He Y, Li Y, Chen C, Zhang B (2020). Association between aspirin use and risk of aneurysmal subarachnoid hemorrhage: A meta-analysis. World Neurosurg..

[17] Florez WA (2021). Relationship between aspirin use and subarachnoid hemorrhage: A systematic review and meta-analysis. Clin. Neurol. Neurosurg..

[18] Ewbank F, Birks J, Bulters D (2022). A meta-analysis of aspirin and subarachnoid haemorrhage in patients with intracranial aneurysms yields different results to the general population. Int. J. Stroke.

[19] Hasan DM (2011). Aspirin as a promising agent for decreasing incidence of cerebral aneurysm rupture. Stroke.

[20] Gross BA, Rosalind Lai PM, Frerichs KU, Du R (2014). Aspirin and aneurysmal subarachnoid hemorrhage. World Neurosurg..

[21] Hostettler IC (2018). Characteristics of unruptured compared to ruptured intracranial aneurysms: A multicenter case-control study. Clin. Neurosurg..

[22] Can A (2018). Association between aspirin dose and subarachnoid hemorrhage from saccular aneurysms: A case-control study. Neurology.

[23] Nisson PL, Meybodi T, Lawton MT, Secomb TW, Roe DJ, Berger GK (2020). Patients taking antithrombotic medications present less frequently with ruptured aneurysms. World Neurosurg..

[24] Weng JC (2021). Safety of aspirin use in patients with stroke and small unruptured aneurysms. Neurology.

[25] Iso H (1999). Prospective study of aspirin use and risk of stroke in women. Stroke.

[26] Schmidt M, Johansen MB, Lash TL, Christiansen CF, Christensen S, Sorensen HT (2010). Antiplatelet drugs and risk of subarachnoid hemorrhage: A population-based case-control study. J. Thromb. Haemost..

[27] Garbe E, Behr S, Kreisel SH (2013). Risk of subarachnoid hemorrhage and early case fatality associated with outpatient antithrombotic drug use. Stroke.

[28] Pottegard A, Hallas J, Rodriguez LAG, Poulsen FR, Gaist D (2015). Antithrombotic drugs and subarachnoid haemorrhage risk: A nationwide case-control study in Denmark. Thromb. Haemost..

[29] Cea SL, Gaist D, Soriano-Gabarró M, Bromley S, García Rodríguez LA (2017). Low-dose aspirin and risk of intracranial bleeds: An observational study in UK general practice. Neurology.

[30] García-Rodríguez LA, Gaist D, Morton J, Cookson C, González-Pérez A (2013). Antithrombotic drugs and risk of hemorrhagic stroke in the general population. Neurology.

[31] Dawber TR, Meadors GF, Moore FE (1951). Epidemiological approaches to heart disease: The Framingham study. Am. J. Public Health Nations Health.

[32] Kannel WB, Feinleib M, McNamara PM, Garrison RJ, Castelli WP (1979). An investigation of coronary heart disease in families. The Framingham offspring study. Am. J. Epidemiol..

[33] Vlak MH, Algra A, Brandenburg R, Rinkel GJ (2011). Prevalence of unruptured intracranial aneurysms, with emphasis on sex, age, comorbidity, country, and time period: A systematic review and meta-analysis. Lancet Neurol..

